# Real-world data on the efficacy and toxicity of induction chemotherapy in locally advanced nasopharyngeal carcinoma in a non-endemic population

**DOI:** 10.3332/ecancer.2025.1832

**Published:** 2025-01-23

**Authors:** Cassio Murilo Hidalgo Filho, Gabriel Berlingieri Polho, Otavio Augusto Moreira, Matheus de Oliveira Andrade, Vinicius Cruz Parrela, Yumi Ricucci Shinkado, Amanda Acioli de Almeida Robatto, Felippe Lazar Neto, Ana Julia Freitas, Aurelio Teixeira Souza, Gilberto de Castro Junior, Milena Perez Mak

**Affiliations:** 1 Department of Clinical Oncology, Instituto do Cancer do Estado de São Paulo ICESP, Hospital das Clinicas HCFMUSP, Faculdade de Medicina, Universidade de São Paulo, São Paulo, SP 05508-220, Brazil; 2 Barretos Cancer Hospital, São Paulo, SP 14784-400, Brazil; 3 Department of Radiotherapy, Instituto do Cancer do Estado de São Paulo ICESP, Hospital das Clinicas HCFMUSP, Faculdade de Medicina, Universidade de São Paulo, São Paulo, SP 05508-220, Brazil; a https://orcid.org/0000-0002-7046-0059

**Keywords:** nasopharynx, carcinoma, chemotherapy, real-world data, LMIC

## Abstract

**Background:**

Induction chemotherapy (ICT) is critical for managing locally advanced nasopharyngeal carcinoma (LA-NPC), but real-world data on its efficacy and toxicity are limited.

**Methods:**

This retrospective study included LA-NPC patients treated with ICT from 2012 to 2022. We evaluated radiological response rates, overall survival (OS), treatment-related toxicities and complete response (CR) rates.

**Results:**

Among 217 patients, 119 met the inclusion criteria and were included in the final analysis. CR rates were similar across ICT regimens (docetaxel, cisplatin and 5-fluorouracil 68.0%; cisplatin and gemcitabine 57.1%; cisplatin and 5-fluorouracil 58.0%; others 50%, *p* = 0.72). Serious adverse events (SAEs) occurred in 22%, with 69.7% experiencing weight loss and 31.9% requiring enteral tube placement. Poor OS was linked to Eastern Cooperative Oncology Group performance status (ECOG-PS) ≥2 hazard ratios (HR 2.8, *p* = 0.004) and residual disease (RD) (HR 7.4, *p* = 0.001). Stage IV (Odds Ratio [OR] 3.77, p = 0.005) and ECOG-PS ≥ 2 (OR 4.69, *p* = 0.006) were associated with RD.

**Conclusion:**

ICT regimens had similar CR rates. Poor ECOG-PS and stage IV predicted RD. Managing toxicities is crucial for better outcomes.

## Introduction

Nasopharyngeal carcinoma (NPC) stands as a unique and challenging entity within the spectrum of head and neck malignancies, characterised by its distinct epidemiological patterns, etiological factors and clinical behaviour [1]. Recently, there has been a growing interest in exploring novel therapeutic strategies to improve outcomes. Induction chemotherapy (ICT) is considered the standard of care for patients with locally advanced disease [2].

ICT, as a therapeutic strategy, holds an essential role in the management of locally advanced nasopharyngeal carcinoma (LA-NPC). By administering systemic chemotherapy before definitive local treatments, such as radiotherapy (RT) or concurrent chemoradiotherapy (CRT), ICT aims to reduce tumour burden, eradicate micrometastases and enhance the overall efficacy of subsequent treatments. The rationale behind this approach lies in its potential to target both the primary tumour and disseminated disease, ultimately optimising the chances of locoregional control and distant metastasis prevention. Additionally, response to ICT predicts the benefit of definitive local treatment [3, 4].

The choice of ICT regimen is influenced by both efficacy and toxicity considerations [5, 6]. Notably, comprehensive trials directly comparing the outcomes of different ICT regimens are scarce. Thus, selecting the most appropriate ICT becomes challenging for clinicians managing LA-NPC cases. There is a need for more evidence-based guidance in this aspect, urging for trials that systematically assess and compare various ICT regimens [7].

Currently, there is limited data on the outcomes of the different ICT options available for NPC among patients in Latin America. This study addresses this gap by evaluating the outcomes of treated NPC patients with ICT at a tertiary cancer center in Brazil, emphasising the differences in efficacy and toxicity profiles among different ICT regimens.

## Materials and methods

### Study design and population

This retrospective cohort study included patients diagnosed with LA-NPC at Instituto do Cancer do Estado de São Paulo, Brazil, a large tertiary cancer center in Latin America, from January 2012 to December 2022. We used the International Classification of Diseases (ICD-10) ‘C11’ criteria to identify those patients with NPC. We included patients who received at least one cycle of planned ICT for LA-NPC. Exclusion criteria comprised (i) de novo metastatic NPC, (ii) concurrent head and neck malignant lesions, (iii) neuroendocrine or small cell carcinoma histologies and (iv) induction treatment information unavailable (e.g., treated outside the institution).

### Data collection

Data for this retrospective cohort study were gathered from electronic health records. The collected data were anonymised and securely stored in REDCap, United States. The extracted variables encompassed demographic and clinical parameters, including sex, age at diagnosis, patient-reported race, Eastern Cooperative Oncology Group performance status (ECOG-PS) at the time of diagnosis, histology, Epstein-Barr virus (EBV) tumour positivity and tobacco exposure (considered present if the patient reported more than 100 cigarettes over their lifetime), body mass index at diagnosis. Moreover, TNM staging (AJCC 8th edition) and detailed information about the treatment, such as the ICT regimen chosen by the physician, the number of chemotherapy cycles, concurrent CRT regimen and RT doses, when available. During the study period, Image-Guided Intensity Modulated Radiation Therapy was consistently applied to all patients receiving RT. The occurrences of serious adverse events (SAEs) during ICT and CRT following the initial ICT were evaluated. SAEs were considered events during ICT or CRT that led patients to hospitalisation or death.

The dataset was enriched with information regarding the type of recurrence (locoregional, distant or both) and toxicities experienced during the treatment, such as (i) enteral feeding (any time of the treatment), (ii) severe mucositis (defined as mucositis interfering oral intake), (iii) weight loss of ≥10% of the baseline, (iv) increase in creatinine levels for more than >2 mg/dL of the baseline and (v) incidence of febrile neutropenia. The last follow-up date for each patient was determined as the most recent contact with the healthcare provider and was updated on 11 March 2024. Hospital staff diligently documented deaths outside the hospital's medical records, ensuring a comprehensive and accurate representation of patient outcomes.

### Outcomes

The primary objectives of this study were to describe the efficacy and explore outcomes related to the toxicity of different ICT regimens for patients with LA-NPC. Efficacy was measured through comprehensive radiological and clinical evaluations of treatment responses, while toxicity profiles were analyzed to understand the safety implications of each regimen.

Additionally, secondary outcomes included overall survival (OS) and recurrence-free survival (RFS) by ICT type. Also, the detailed description of definitive therapy (CRT or exclusive RT) after ICT and the attainment of complete radiological response (CR) or residual disease (RD) across different treatment groups. All imaging studies were reviewed by radiologists specialised in head and neck cancers. Head and neck cancer oncologists retrospectively evaluated the radiological response. The first image (computed tomography or magnetic resonance imaging) within at least 8–12 weeks after the completion of CRT was considered for response evaluation.

### Statistical analysis

Descriptive statistics were employed to characterise the numeric variables. Measures such as mean, standard deviation, median or interquartile range (IQR) were utilised based on the distribution of each variable. Categorical variables were presented using absolute numbers and proportions, expressed as percentages and compared with the Fisher and chi-square tests. Logistic regression tested the relation between the CR rate and different independent variables. Only statistically significant variables were included in the multivariate analysis. Mortality rates were estimated for the overall patient cohort, among different induction therapy regimens and between patients with and without recurrence. RFS was calculated from the first ICT doses to the occurrence of a recurrence confirmed by imaging. OS was calculated from the date of the first chemotherapy doses to the occurrence of death or the last follow-up. In cases where no event occurred, patients were censored at the last follow-up date. Hazard ratios (HRs) for OS were calculated using Cox proportional hazard models. Survival and time probabilities were estimated using the Kaplan-Meier method. The statistical analyses were executed utilising R for Statistical Computing (Vienna, Austria) version 4.2.1 (2022-06-23).

### Ethical approval

This study received ethical approval from the local ethics committee, under approval number 49094921.4.0000.0068, in compliance with ethical standards and patient confidentiality.

## Results

### Patients' characteristics

**Supplementary Figure S1.**
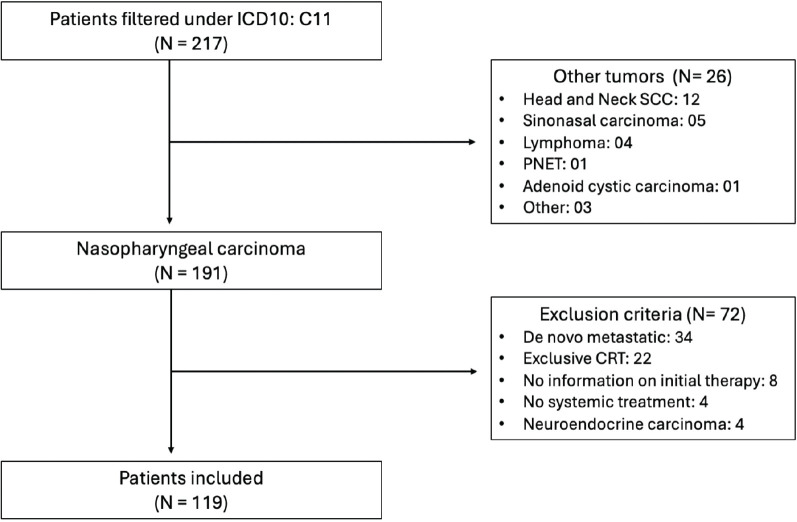
Flow chart of patients included in the study.

A total of 217 patients were initially identified through the ICD filter. After applying the study criteria, 119 patients were included in the final analysis (Supplementary Figure S1). Among the included patients, the median age was 49.5 years old (IQR 29.5–58.7). Most (59.7%) were male and self-referred as white (74%). The majority had ECOG-PS 0-1 (84.7%) and stage IV disease (71.4%).

The most prevalent histology in the cohort was undifferentiated non-keratinising carcinoma (WHO type 3), corresponding to 58% of the patients, followed by keratinising squamous cell carcinoma (WHO type 1) (33%). The EBV status was tested by *in situ* hybridisation in 62% and found positive in 55 patients, corresponding to 46% of the entire cohort. Detailed patient characteristics are presented in Table 1.

### Treatment details

The most prescribed ICT regimen was cisplatin and gemcitabine (CG), followed by cisplatin and 5-fluorouracil (CF) and docetaxel, cisplatin and 5-fluorouracil (TPF) (41%, 26% and 21%, respectively). Among other regimens of induction therapy by physician’s choice, seven patients received carboplatin and paclitaxel (CP) (5.8%), five carboplatin and 5-fluorouracil (4.2%) and two patients received CP followed by CG (Table 2). Most of our cohort underwent concurrent CRT (89%) with cisplatin 100 mg per m^2^ (mg/m^2^) every 3 weeks (98%). Only two patients received cisplatin 40 mg/m^2^ weekly.

RT alone was performed for nine patients due to poor performance status after induction therapy. Four patients received only one cycle of ICT and no further treatment. Three of them died after the ICT, two due to rapid progressive cancer and one due to sepsis. One patient lost follow-up after one cycle. The median RT duration was 7.0 weeks (IQR 6.57–7.86), while approximately 90% received more than 6 weeks of CRT and the planned dosing of 7000 cGY.

Among the patients who did not complete the full course of CRT, the main reason was toxicity, especially during the last third of the treatment course. Of the 44 patients who did not complete CRT, 33 (75%) stopped due to toxicity, 8 (18.2%) were lost to follow-up or had treatment compliance issues, 2 (4.5%) chose not to continue treatment and 1 (2.3%) discontinued due to disease progression.

Patients with ECOG PS ≥2 (*n* = 17) experienced significantly lower treatment compliance compared to the overall cohort. Only 29.4% completed the full course of CRT. Notably these patients also had more advanced disease at the diagnosis (Supplementary Table S1).

### Toxicity profile

SAEs leading to hospitalisation occurred in 22% of the entire cohort. During the treatment, 69.7% of the patients experienced weight loss ≥10% of the baseline. Mucositis leading to decreased oral intake occurred in 53.8%, and 31.9% required an enteral tube placement for feeding during the treatment. In addition, increased creatinine levels (10.1%) or febrile neutropenia (5.9%) were also reported. There were no statistically significant differences in toxicities among different ICT regimens. However, CF was associated with numerically more weight loss (80.6%) and enteral feeding (34.5%) than other ICT choices. CG was associated with numerically less mucositis (42.8% versus 64% versus 61.2%) and enteral feeding than TPF and CF (26.5% versus 32% versus 35.4%). Despite the distinct toxicity profiles, CRT completion rates were similar among different induction regimens (*p* = 0.33). The details of treatment toxicity are described in Table 2.

### Patients’ outcomes

**Supplementary Figure S2.**
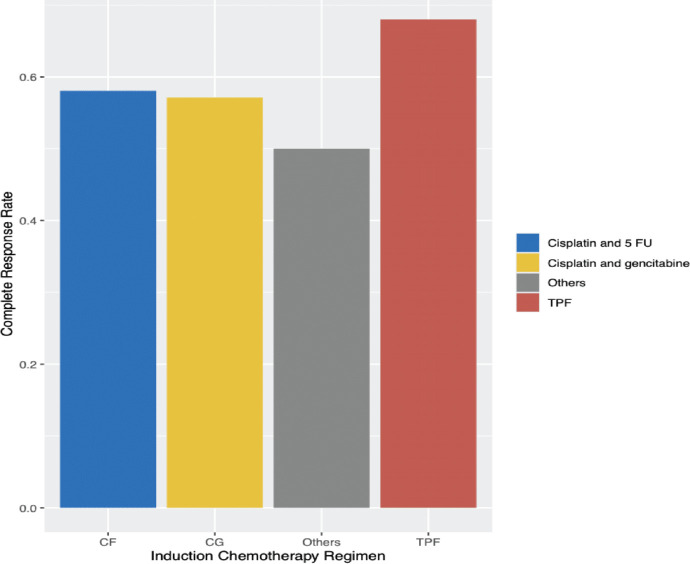
Radiological CR rate by ICT regimen among patients with LA-NPC. CRT: chemoradiotherapy, TPF: docetaxel, cisplatin and 5-fluorouracil, CG: Cisplatin + Gemcitabine, CF: Cisplatin + 5-fluorouracil.

Most patients achieved radiological response (Table 3). Radiological CR rates were similar among ICT regimens (TPF 68.0%; CG 57.1%; CF 58.0%; others 50%, *p* = 0.72) (Supplementary Figure S2). There was no difference in CR rate among different histologies (*p* = 0.75), completion of CRT (*p* = 0.83) or EBV status (*p* = 0.18). In the univariate logistic regression, factors that increased the odds of RD after ICT followed by a definite treatment were: Stage IV (Odds Ratio (OR) 3.77, 95% CI 1.54–10.2, *p* = 0.005), SAEs (OR 3.19, 95% CI 1.32–8.00, *p* = 0.01) and ECOG-PS ≥2 (OR 4.69 95% CI 1.63–15.6, *p* = 0.006). There was no difference in CR rate between keratinising versus non-keratinising histologies, completion of CRT or EBV positivity status (Table 4). In multivariate analysis, ECOG-PS ≥2 (OR 3.96, 95% CI 1.33–13.4, *p* = 0.017) and stage IV (OR 3.29, 95% CI 1.32–9.11, *p* = 0.014) remained significant.

**Figure 1.**
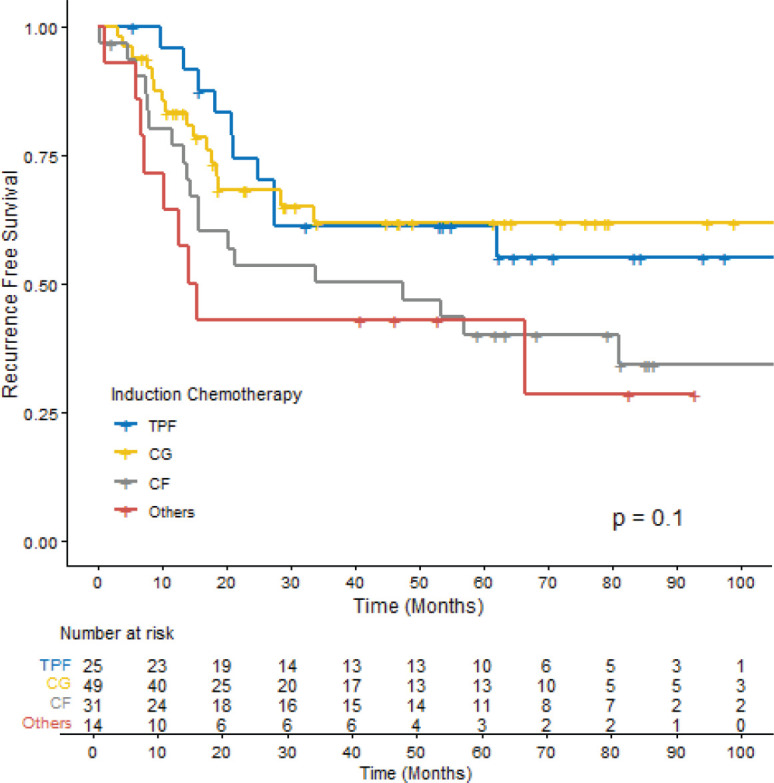
RFS according to ICT among patients with LA-NPC. TPF: docetaxel, cisplatin and 5-fluorouracil, CG: Cisplatin + Gemcitabine, CF: Cisplatin + 5-fluorouracil.

Among the patients who experienced disease recurrence (36.9%), distant recurrence was more common (63.6%) than locoregional recurrence (29.5%). Additionally, a small proportion of patients had both distant and locoregional recurrences (6.8%). The RFS rates varied among the ICT regimens. The 12-month RFS was 95.3% (95% CI 88.1–100.0) for TPF, 83.2% (95% CI 73.2–94.5) for CG, 76.8% (95% CI 63.0–93.4) for CF and 64.3% (95% CI 43.5–95.0) for other regimens. However, no statistical difference was observed among them (*p* = 0.1) (Figure 1).

**Figure 2.**
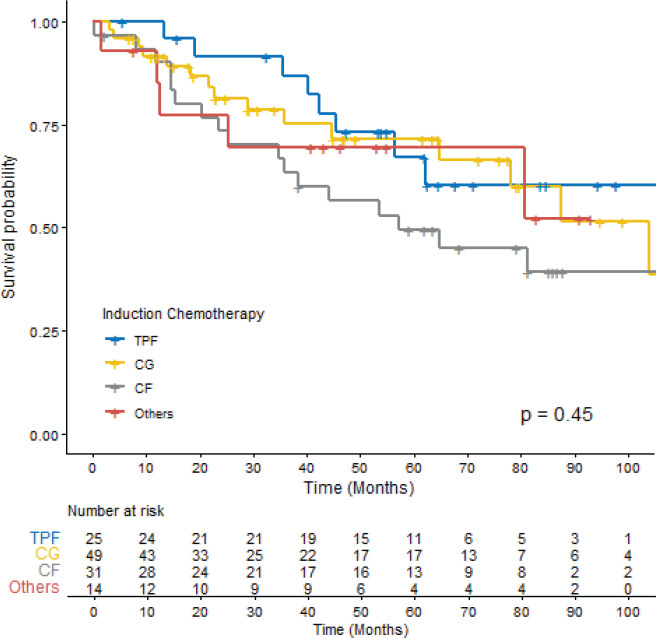
OS according to ICT among patients with LA-NPC. TPF: docetaxel, cisplatin and 5-fluorouracil, CG: Cisplatin + Gemcitabine, CF: Cisplatin + 5-fluorouracil.

The OS for the entire cohort was 87.5 months (CI 95% 64.9–122.0). The median follow-up time was 64.6 months. In the univariate Cox regression analysis, ECOG-PS ≥2 (*p* = 0.004) and incomplete radiological response (*p* < 0.001) were associated with lower OS rates. However, no significant OS differences were observed based on the chemotherapy regimen (Figure 2, *p* = 0.45), the occurrence of SAEs (*p* = 0.2) or the completion of CRT (*p* = 0.8) (Table 5).

## Discussion

In this study, we provided data about the outcomes of patients with LA-NPC who underwent ICT in a large cancer center in Brazil and the largest cohort described in Latin America. There was no difference in survival or toxicities among different ICT regimens. Patients with poor ECOG-PS experienced worse outcomes. In addition, ECOG-PS ≥2, stage IV and the occurrence of SAEs were associated with RD after ICT followed by a definitive treatment, including CRT or isolated RT. CG regimen showed a more favourable toxicity profile, including less mucositis and a lower rate of enteral tube placement during the treatment.

The current standard of care for patients with LA-NPC includes CRT in combination with induction therapy or CRT, followed by adjuvant chemotherapy [8]. Currently, no randomised trials comparing both strategies have been performed. Previous phase III clinical trials have tested various ICT regimens (CF, CG or TPF) in combination with CRT, each compared to CRT alone and demonstrated improved survival [9–11]. However, clinical trials have mainly included patients from Asia, where NPC is endemic. There is little representativeness of the Latin American population in NPC trials evaluating induction systemic therapy. Although a low incidence of this malignancy is observed in Latin America, recent data indicated that the incidence and mortality of NPC have been increasing in low and middle-income Latin American nations, especially Brazil, in the last three decades [12, 13]. Also, few retrospective series have addressed the outcomes of Brazilian patients diagnosed with NPC [14, 15]. In our cohort, despite nonkeratinising carcinoma (WHO type 3) being the most prevalent histology, keratinising squamous cell carcinoma (WHO type 1) corresponded to a representative number, one-third of the cases, which was a higher proportion than what was observed in Finland [16] and the Netherlands [17], but similar to a previous report of a national database in the United States [18]. There is a low number of patients tested for EBV positivity in the tumour, and it was positive for approximately 72% of patients with available EBV status. Our data suggests that the EBV positivity status was lower than previous data from another Brazilian study [19]. Finally, our study differs from other cohorts in Latin America by including only LA-NPC patients and evaluating the outcomes related to each ICT regimen.

The current optimal ICT regimen still needs to be clarified, and oncologists base their therapy choice on each combination's efficacy and toxicity profile [20]. Our study shows that most patients experienced significant weight loss, as observed in other studies evaluating patients with similar therapies [21]. We also reported that severe mucositis (53.8%) and enteral feeding (31.9%) were relatively common findings, supporting previous data acknowledging RT-induced mucositis's association with malnutrition and weight loss [22–24]. Moreover, our real-world data reveal substantial toxicity rates, which are often higher than those reported in clinical trials due to the controlled environments and stringent patient selection criteria of trials. We observed significant rates of weight loss, mucositis and the need for enteral feeding during treatment, emphasising the importance of proactive management strategies to mitigate treatment-related adverse events. These findings underscore the multidisciplinary nature of NPC care. Effective supportive care interventions are crucial for optimising treatment outcomes in the real-world setting.

It is essential to acknowledge the limitations of our study. First, it was conducted retrospectively, which may introduce biases and limit the ability to draw causal conclusions. Furthermore, in the context of global oncology, access to certain diagnostic tests, such as EBV status, may be limited and as a result, EBV testing was not available for all patients in our study, despite its known strong association with NPC. However, diagnoses were made by a specialised multidisciplinary team, including pathologists, radiologists, head and neck surgeons and oncologists, which likely ensured accuracy. The retrospective collection of toxicity profiles from medical records may have resulted in underreporting of adverse events, which could affect the overall interpretation of our findings. Detailed adverse event data, such as those classified under CTCAE, were not consistently available, which is usually a limitation of retrospective studies. Finally, the response evaluation was performed at distinct times and with different radiological methods (computed tomography scans or magnetic resonance imaging), and positron emission tomography – computed tomography scan was not routinely used for response evaluation. Nevertheless, our study provides valuable real-world data to the limited body of literature on NPC management in a non-endemic country, highlighting the need for further research to refine treatment strategies and improve patient outcomes in this region.

## Conclusion

In conclusion, this study suggests no significant differences in CR rates or survival outcomes among different ICT regimens in the studied population. Patients with distant recurrence and poor ECOG-PS experienced worse survival outcomes. The study suggests that ECOG-PS ≥2, SAEs and stage IV are associated with a lower chance of CR, emphasising the importance of early detection and effective management of treatment adverse events. The study also provides valuable insights into the toxicity profiles of different ICT regimens, highlighting the need for multidisciplinary approaches to manage treatment-related events effectively. Overall, the study contributes to understanding NPC management and underscores the importance of further research to optimise treatment strategies and improve patient outcomes.

## Conflicts of interest

**Cassio Murilo Hidalgo Filho, Matheus de Oliveira Andrade, Vinicius Cruz Parrela, Yumi Ricucci Shinkado, Amanda Acioli de Almeida, Felippe Lazar Neto, Ana Julia Freitas**, **Aurelio Teixeira Souza**: Declare no conflict of interest.

**Otavio Augusto Moreira:**

**Honoraria**: Bayer, Novartis, Astrazeneca.

**Gilberto de Castro Junior**: **Honoraria:** AstraZeneca, Pfizer, Merck Sharp & Dohme, Bristol Myers Squibb, Novartis, Roche, Amgen, Janssen, Merck Serono, Lilly, Takeda, Daiichi Sankyo/UCB Japan

**Consulting or Advisory Role:** Boehringer Ingelheim, Pfizer, Bayer, Roche, Merck Sharp & Dohme, Bristol Myers Squibb, AstraZeneca, Yuhan, Merck Serono, Janssen, Libbs, Sanofi, Novartis, Lilly, Takeda, Daiichi Sankyo/UCB Japan

**Speakers' Bureau:** AstraZeneca, Bayer, Novartis, Roche, Merck Serono, Bristol Myers Squibb, Merck Sharp & Dohme, Boehringer Ingelheim, Pfizer, Janssen, Amgen, Takeda

**Travel, Accommodations, Expenses:** Merck Sharp & Dohme, Novartis, Pfizer, Roche, AstraZeneca, Boehringer Ingelheim, Bayer, Bristol Myers Squibb, Merck Serono, Daiichi Sankyo/UCB Japan

**Milena Perez Mak:**

**Honoraria:** Bayer, Pfizer, Merck Serono, Takeda, Amgen Consulting or Advisory Role: AstraZeneca.

## Funding

No funding was received for this study.

## Author contributions

**Cassio Murilo Hidalgo Filho:** Conceptualisation, methodology, project administration, supervision, data collection, visualisation, formal analysis, writing original draft, writing review and editing. **Gabriel Berlingieri Polho:** Methodology, project administration, supervision, data collection, visualisation, formal analysis, writing original draft, writing review and editing.

**Matheus de Oliveira Andrade, Vinicius Cruz Parrela, Yumi Ricucci Shinkado, Amanda Acioli de Almeida:** Supervision, data collection, visualisation, writing review and editing. **Felippe Lazar Neto:** Supervision, visualisation, formal analysis, writing review and editing. **Ana Julia Freitas:** Methodology, formal analysis, writing review and editing. **Aurelio Teixeira Souza:** Supervision, visualisation, writing review and editing.** Gilberto de Castro Junior**: Conceptualisation, methodology, project administration, supervision, visualisation, writing review and editing. **Milena Perez Mak:** Conceptualisation, methodology, project administration, supervision, visualisation, writing review and editing.

## Figures and Tables

**Table 1.** Clinical characteristics of patients diagnosed with LA-NPC treated with ICT.

Characteristic	Mean	Median	IQR	Percentile 25	Percentile 75	SD
**Age**	45.83	49.52	29.27	29.5	58.77	16.65
**Smoking history (pack/years)**	32.38	20	33	15	48	24.55
Characteristic					n	%
**Sex**	Male				71	59.67
	Female				48	40.33
**Race**	White				88	73.94
	Brown/Black				25	21.00
	Yellow				2	1.68
	Not informed				4	3.38
**Histology**	Undifferentiated non-keratinizing carcinoma				70	58.82
	Keratinizing squamous cell carcinoma				40	33.61
	Differentiated non-keratinizing carcinoma				6	5.04
	Unavailable				3	2.53
**EBV**	Positive				55	46.21
	Negative				20	16.80
	Inconclusive				1	0.85
	Unavailable				43	36.14
**Smoking**	No				64	53.78
	Yes				55	46.22
**ECOG-PS**	0				33	27.73
	1				68	57.14
	2				14	11.76
	3				3	2.52
	Unavailable				1	0.85
**TNM staging**	III				34	28.58
	IV				85	71.42
**ICT regimen**	CG				49	41.17
	CF				31	26.05
	TPF				25	21.00
	CP				7	5.90
	Carboplatin + 5FU				5	4.20
	Other				2	1.68
**Subsequent therapy**	CRT				106	89.08
	RT				9	7.56
	None				4	3.36
**Concurrent Chemotherapy**	Cisplatin 100 mg q3w ×3				104	98.11
	Cisplatin 40 mg q1w ×6				2	1.88

LA-NPC: Locally advanced nasopharyngeal carcinoma, ICT: induction chemotherapy, SD: standard deviation, ECOG: Eastern Cooperative Oncology Group performance status, CRT: chemoradiotherapy, RT: radiotherapy, TPF: docetaxel, cisplatin and 5-fluorouracil, CG: Cisplatin + Gemcitabine, CF: Cisplatin + 5-fluorouracil

**Table 2.** Descriptive table of toxicity for patients diagnosed with LA-NPC by each ICT regimen.

	TPF (%) *N* = 25	CG (%) *N* = 49	CF (%) *N* = 31	Other (%) *N* = 14	All (%) *N* = 119	*p* value
Weight loss ≥10%						
Yes	17 (68.00)	33 (67.35)	25 (80.65)	8 (57.14)	83 (69.70)	0.37
No	8 (32.00)	16 (32.65)	6 (19.35)	6 (42.86)	36 (30.30)	
Severe mucositis						
Yes	16 (64.00)	21 (42.86)	19 (61.29)	8 (57.14)	64 (53.80)	0.25
No	9 (36.00)	28 (57.14)	12 (38.71)	6 (42.86)	55 (46.20)	
Enteral feeding						
Yes	8 (32.00)	13 (26.53)	11 (35.48)	6 (42.86)	38 (31.90)	0.64
No	17 (68.00)	36 (73.47)	20 (64.52)	8 (57.14)	81 (68.10)	
Increase in creatinine*						
Yes	0 (0.00)	7 (14.29)	3 (9.68)	2 (14.29)	12 (10.10)	0.20
No	25 (100.00)	42 (85.71)	28 (90.32)	12 (85.71)	107 (89.90)	
Febrile neutropenia						
Yes	1 (4.00)	3 (6.12)	3 (9.68)	0 (0.00)	7 (5.90)	0.73
No	24 (96.00)	46 (93.88)	28 (90.32)	14(100.00)	112(94.10)	
SAEs						
Yes	5 (20.00)	9 (18.37)	7 (22.58)	6 (42.86)	27 (22.70)	0.29
No	20 (80.00)	40 (81.63)	24 (77.42)	8 (57.14)	92 (77.30)	

* Increase in creatinine levels ≥2 mg/dL

ICT: Induction chemotherapy, TPF: docetaxel, cisplatin and 5-fluorouracil, CG: Cisplatin + Gemcitabine, CF: Cisplatin + 5-fluorouracil

**Table 3.** Outcomes of patients diagnosed with LA-NPC treated with ICT.

Outcomes		*n*	%
Treatment response	PR	32	26.90
	CR	70	58.82
	SD	2	1.68
	PD	7	5.88
	Unavailable	8	6.72
Complete CRT	No	44	41.90
	Yes	62	58.10
Reason for incomplete CRT*	Toxicity	33	75
	Death	1	2.28
	Lost follow-up	3	6.82
	Other	2	4.54
	Unavaliable	5	11.36
Recurrence	No	75	63.02
	Yes	44	36.98
Recurrence type	Distance	28	63.64
	Locorregional only	13	29.54
	Both	3	6.82


LA-*Among patients who did not complete the chemoradiotherapy treatment

NPC: Locally advanced nasopharyngeal carcinoma, ICT: induction chemotherapy, ECOG: Eastern Cooperative Oncology Group performance status, CRT: chemoradiotherapy, RT: radiotherapy, PR: partial response, CR: complete response, SD: stable disease, PD: progressive disease

**Table 4.** Univariate analysis of patients with LA-NPC with RD after ICT and CRT or RT.

Variable	% RD	OR	CI 95%	*p*-value
Stage IV	49.4	3.77	1.54–10.2	0.005
TPF	32	1		
CG	42.9	1.59	0.58–4.55	0.37
CF	42	1.53	0.51–4.75	0.44
Others	50	2.12	0.55–8.40	0.27
EBV negative	35	1	-	-
EBV positive	34.6	0.98	0.34–2.99	0.97
EBV unknow	52.2	0.53	0.69–6.33	0.20
Occurrence of SAE	63	3.19	1.33–8.01	0.01
ECOG-PS ≥ 2	72.2	4.69	1.63–15.61	0.006
Keratinizing histology	42.5	1	-	-
Non-keratinizing histology	40.5	0.92	0.43–2.01	0.9

OR: Odds ratio, LA-NPC: Locally advanced nasopharyngeal carcinoma, RD: residual disease, ICT: induction chemotherapy, CRT: chemoradiotherapy, EBV: Epstein Barr virus, ECOG: Eastern Cooperative Oncology Group performance status, TPF: docetaxel, cisplatin and 5-fluorouracil, CG: Cisplatin + Gemcitabine, CF: Cisplatin + 5-fluorouracil, SAE: Serious adverse event

**Table 5.** Univariate Cox regression for variables associated with OS among patients with LA-NPC.

Chemotherapy regimen	HR (CI 95%)	*p*-value
TPF	Ref	0.5
CG	1.2 (0.5–2.8)	-
CF	1.8 (0.8–4.3)	-
Other	1.3 (0.4–4.0)	-
ECOG ≥ 2	2.8 (1.4–5.7)	0.004
RD	7.4 (3.8–14.3)	<0.001
Recurrence	6.8 (3.4–13.4)	<0.001
SAEs	1.5 (0.79–3.0)	0.2
Incomplete CRT	1.1 (0.54–2.1)	0.8

HR: Hazard ratio, ECOG: Eastern Cooperative Oncology Group performance status, TPF: docetaxel, cisplatin and 5-fluorouracil, CG: Cisplatin + Gemcitabine, CF: Cisplatin + 5-fluorouracil, CRT: chemoradiotherapy

## Supplementary material

**Supplementary Table S1.** Patients with ECOG-PS ≥2 demographic characteristics and treatment details.

Characteristic		*n*	%
Staging	IV	15	88.24
	III	2	11.76
Sex	Male	14	82.35
	Female	3	17.65
Race	White	11	64.70
	Other	6	35.30
Histology	Keratinizing squamous cell carcinoma	10	58.82
	Undifferentiated nonkeratinizing carcinoma	7	41.18
EBV	Positive	6	35.30
	Negative	2	11.76
	Unavailable	9	52.94
Tabagism	Yes	10	58.82
	No	7	41.18
Treatment performed	CRT	10	58.82
	RT monotherapy	3	17.65
	ICT only*	4	23.53
Recurrence	Yes	9	52.94
	No	8	47.06
Death	Yes	10	58.82
	No	7	41.18

* Three patients died during ICT and one patient lost follow-up after first cycle of ICT

ECOG: Eastern Cooperative Oncology Group performance status, ICT: induction chemotherapy, CRT: chemoradiotherapy, RT: radiotherapy, EBV: Epstein Barr virus
